# An Accurate FFPA-PSR Estimator Algorithm and Tool for Software Effort Estimation

**DOI:** 10.1155/2015/919825

**Published:** 2015-05-20

**Authors:** Senthil Kumar Murugesan, Chidhambara Rajan Balasubramanian

**Affiliations:** ^1^Department of Computer Science and Engineering, Valliammai Engineering College, Kattankulathur, Tamil Nadu 603203, India; ^2^Department of Electronics and Communication Engineering, Valliammai Engineering College, Kattankulathur, Tamil Nadu 603203, India

## Abstract

Software companies are now keen to provide secure software with respect to accuracy and reliability of their products especially related to the software effort estimation. Therefore, there is a need to develop a hybrid tool which provides all the necessary features. This paper attempts to propose a hybrid estimator algorithm and model which incorporates quality metrics, reliability factor, and the security factor with a fuzzy-based function point analysis. Initially, this method utilizes a fuzzy-based estimate to control the uncertainty in the software size with the help of a triangular fuzzy set at the early development stage. Secondly, the function point analysis is extended by the security and reliability factors in the calculation. Finally, the performance metrics are added with the effort estimation for accuracy. The experimentation is done with different project data sets on the hybrid tool, and the results are compared with the existing models. It shows that the proposed method not only improves the accuracy but also increases the reliability, as well as the security, of the product.

## 1. Introduction

Software effort estimation plays a vital role in the software project management, since it is the base for many activities like planning, scheduling, and tracking the software projects [1]. The accurate and reliable effort estimation is very important for project managers [2]. If the estimation is not properly calculated then it may result in the failure of the software project [3]. The system security is another important challenge in today's competitive world [4]. The previous research work of the authors showed that performance metrics mainly help in the improvement of the accuracy and the fuzzy-based function point analysis is used to overcome the uncertainty in the effort estimation [5].

One of the most needed requirements in the software company is to estimate the effort in the earlier phase of development process. The various software evaluation metrics and methods are available; as the software systems grow in size, it is really difficult to estimate the effort in accurate way. Additionally, software project estimation is vulnerable to attack. So it is needed to provide a reliable and secure estimation method.

The aim of this work is to provide an estimator model called FFPA-PSR (fuzzy-based function point analysis with performance metrics, security, and reliability factors) which attempts to help in improving the accuracy and reliability of the estimation in secured fashion. This work intends the utilization of concepts from fuzzy logic to classify the attributes in the estimation by framing the new fuzzy rules. This research also introduces the new factors like product security, reliability in the existing value adjustment factors. The performance metric factors are also appended to make the estimation model more accurate automated tool. The main advantage of this tool is to estimate the accurate effort with reliability and security in the automated way. Accurate effort is more important, because most of the software failures in the IT sectors are due to inaccuracy and insecure estimation which leads to huge difference in the expected and actual budget.

## 2. Materials and Methods

There are many fuzzy-based approaches and other related methods which are proposed to estimate the accurate effort. Therefore, only a few approaches are discussed here. The work [6] suggested ambiguous and linguistic inputs of software cost estimation. The work [7] noted that homogeneous data set results in better and more accurate effort estimates, while the irrelevant and disordered data set results in lesser accuracy. The paper [8] proposed a fuzzy logic based framework for managing the imprecision and uncertainty problem. The work [9] proposed a methodology combining the neurofuzzy technique and SEER-SEM that can function with various algorithmic models. The work [10] proposed Enhancing Software Sizing Adjustment Factors. Their results showed that the enhancement achieved good accuracy.

The work [11] proposed an improved analogy-based approach based on extensive dimension weighting. Their results empirically evaluated the accuracy and reliability improvements of the project efforts.

The work [12] suggested that the modification of standard function point complexity weights system can reduce the ambiguity in the effort estimation.

The work [13] demonstrated that the effort estimation done by applying the soft computing technique is powerful in solving real world application with imprecise and uncertain information.

The similarities between these studies are that they all focus on the data sets or the initial phase of the estimation but do not concentrate on the development phase of the effort. Many methods use the fuzzy logic to handle imprecision in the data sets. Some methods concentrate only on accuracy but do not focus on performance factors. Few methods suggested security factor for product security. In this paper, the authors decided to concentrate on all these issues.

## 3. FFPA-PSR Algorithm and Model

The proposed model named as fuzzy-based function point analysis with performance metrics, security, and reliability factors (FFPA-PSR) is based on the FFPA-PSR algorithm which is given in Table 1.

**Figure 1:**
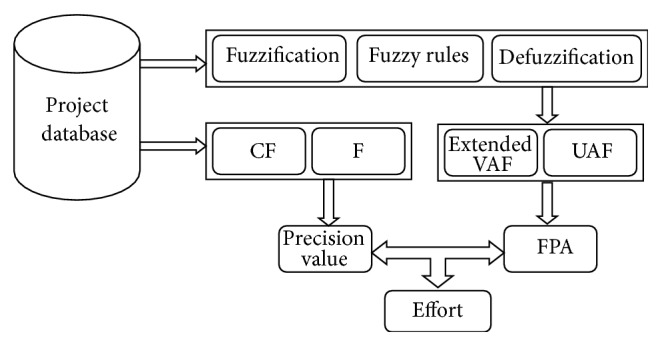
FFPA-PSR estimator tool model.

The structure of the developed model is shown in Figure 1. The input of this model is the software size. The output is the estimated effort of the software. There are four major steps in the estimator model: (1) fuzzy inference system, (2) precision value, (3) extended FPA calculation, and (4) effort estimation.

### 3.1. Fuzzy Inference System

Software development is a very complex process, there are many factors contributing to development effort, and there exists a complex interaction between the factors. In the function point analysis, five factors are used as input named as External Inputs (EI), External Outputs (EO), External Inquiries (EQ), External Interfaces File (EIF), and Internal Logical Files (ILF). Rating of these factors can be given by linguistic terms such as “simple,” “average,” and “complex.” All these factors are fuzzified to handle the imprecision in the data set and need proper handling of the dependencies among these factors to improve the accuracy [13]. Unfortunately, this is not an easy task in most cases, so the authors propose new fuzzy if-then rules to handle this situation:

 
*IF* complexity is* LOW* and the weight is* SMALL,*
 
*THEN* the fuzzy function point is* SIMPLE;*
 
*IF* complexity is AVERAGE and the weight is* MEDIUM,*
 
*THEN* the fuzzy function point is* AVERAGE;*
 
*IF* complexity is HIGH and the weight is* BIG,*
 
*THEN* the fuzzy function point is* COMPLEX.*

The outputs of each fuzzy rule are needed to normalize for the required output. This is done by defuzzification, which converts the fuzzy output into a crisp solution by using the following equation:

(1) $$\begin{array}{c} \text{Output}=\frac{\sum W_{i}*V_{i}}{W_{i}}, \end{array}$$

where *W*_*i*_ = Weighted Average and *V*_*i*_ = Peak Value.

### 3.2. Calculating the Precision Value

Computer performance is characterized by the amount of useful work accomplished by a computer system which is compared to the time and resources used. The performance of any software can be evaluated in measurable, technical terms, using one or more of the following metrics:

- Speed and latency.
- Safety criticalness.
- Precision or accuracy.
- Reliability and availability.
- Robustness or fault-tolerance.
- Capacity.
- Scalability or extensibility.
- Longevity.

The formula to determine the precision value is given below:

(2) $$\begin{array}{c} \text{Precision Value (PV)}=0.01*\left(\;\overset{8}{\underset{i=1}{\sum }}F_{i}*C_{i}\;\right), \end{array}$$

where *F*_*i*_ = factor of each performance metric and *C*_*i*_ = complexity factor.

### 3.3. Effort Calculation

The traditional value adjustment factor calculation has 14 general system characteristics. Now, 15th and 16th factors named product security and product reliability are added to it. So, the formula for calculation of value adjustment factor (VAF) is modified. Consider

(3) VAFTDI∗0.01+0.65,TDI=∑i=116DI.

All the general system characteristic factors including the 15th and 16th factors are rated by the six-point scale (0–5) according to the relevant degree of influence (DI) on application which is given in Table 2.

Then the extended function point count is calculated by multiplying the value adjustment factor with the fuzzified unadjusted function point. Consider

(4) $$\begin{array}{c} \text{EFPA}=\text{UFP}*\text{VAF}. \end{array}$$

Once the precision value and the function point count are calculated, then the enhanced function point count is done by adding the precision value with the extended function point count. Consider

(5) $$\begin{array}{c} \text{FFPA-PSR}=\text{EFPA}+\text{PV}. \end{array}$$

## 4. Results and Discussion

The proposed estimator tool (FFPA-PSR) has been developed in Java script under Windows environment and the validation is done by applying different real project data sets. The effort estimation data of ten software projects implemented in 2013 are used for testing. At the same time, actual effort, traditional function point method, and the authors' previously proposed model called fuzzy-based function point method (FFPA-PM) are also used for comparison with the proposed effort.  Table 3 is the result of the comparison.

**Figure 2:**
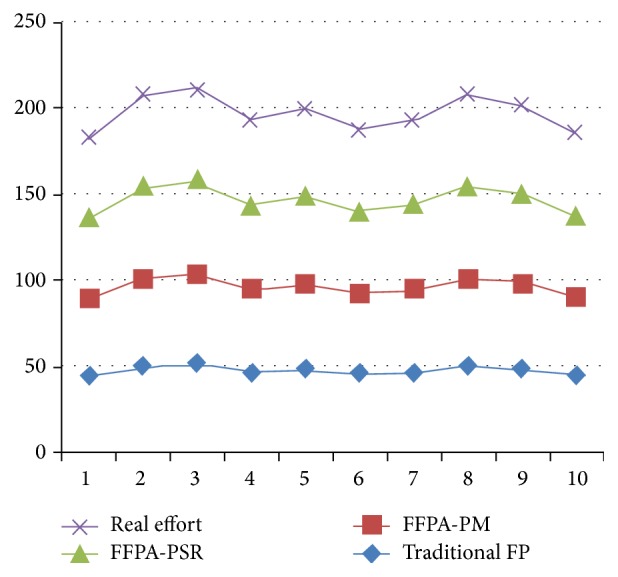
Effort chart.

Figure 2 shows that the proposed method values are very close to the real values. It obviously means that the accuracy of the proposed method is high, but practically it can be evaluated by using the well-known performance evaluation parameters such as MMRE and PRED which are applied to assess as well as to compare the accuracy of the estimated models. Consider

(6) Mean  Relative  Error=Real  Effort−Calculated  EffortReal  Effort.

The Prediction Accuracy (PRED) can be calculated as

(7) $$\begin{array}{c} \text{PRED}=\frac{\lambda }{N}, \end{array}$$

where *λ* is the number of projects and *N* is the number of all estimates.

Table 3 effort values are used to find the MMRE and PRED calculation. The results show that the proposed model performs better than the existing models in terms of accuracy, showing as lower MMRE value and higher prediction value.  Table 4 shows the performance comparison of the average of project data.

**Figure 3:**
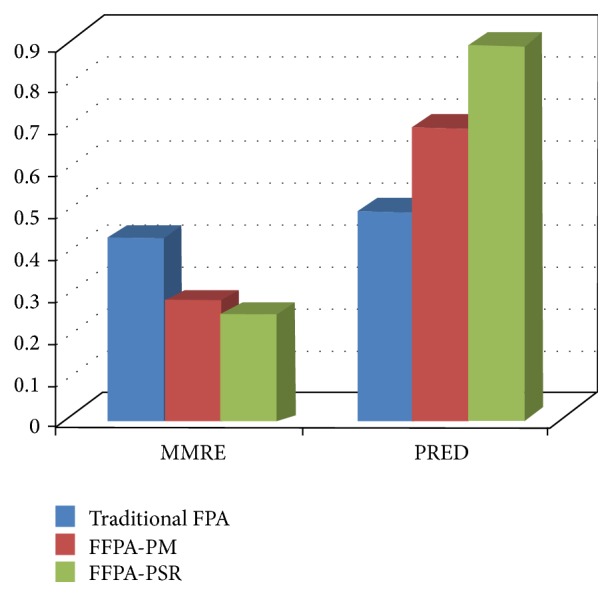
Comparison of the MMRE and PRED.

**Figure 4:**
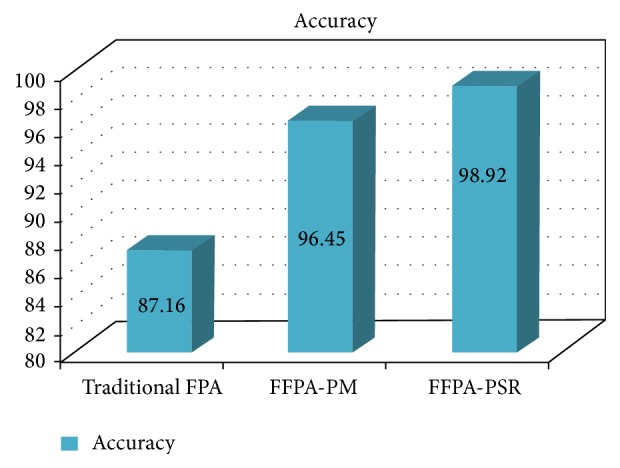
Comparison of accuracy.

In addition, the choice of reliability and security factors has certain influence on the accuracy of the proposed model by 11.76% with the function point model and 2.47% with the authors' previously proposed model (FFPA-PM). Through this experiment and results, it is found that the FFPA-PSR can effectively describe the relative importance of different extended attributes to the FPA, and it significantly enhances the project effort estimation accuracy. Figures 3 and 4 clearly show the improved accuracy of the proposed method in terms of MMRE, PRED, and accuracy.

## 5. Conclusion

Software project development requires the sophisticated methods in helping project managers to manage uncertainty and inaccurate effort estimation. In this paper, it has been shown that the automated hybrid tool can be used to estimate the effort accurately of software projects in secured way. Using a sample data sets integrated by ten different domain projects from different developers, the existing models and proposed model were generated and validated. It can be seen that this proposed model provides better results than the previous existing models. This Proposed Model FFPA-PSR tool which controls the imprecision issues in estimation and also provides a secure and reliable effort estimation which is used in the time critical and mission critical projects. This model also has the ability to handle the incomplete information at the initial stage of the requirement analysis and to determine genuine cause and effect relationship among the factors. The proposed model may also be very useful to estimate the accurate effort within the time and budget frame which is much needed requirement of today's software companies. Therefore, a promising line of future work is to extend this research work by adding risk assessment which helps in risk management of the software projects. This work also can be enhanced by automating the scheduling and tracking of the software projects in the near future.

## Figures and Tables

**Table 1:** FFPA-PSR algorithm.

S. number	Steps	Actions
1	[Start]	Generate the specification of the project
2	[Specify]	Specify the adjusted function point
3	[Classify]	Classify the function point analysis using triangular membership function
4	[Inference]	Cross over the fuzzy rules
5	[Extend]	Extend the VAF with security and reliability factor
6	[Calculate]	Calculate the fuzzy function point by using UFP & VAF
7	[Find]	Find the performance metrics by calculating the precision value
8	[Append]	Append the precision value with FFPA
9	[Estimate]	Calculate the enhanced effort estimation
10	[Test]	Test the accuracy using real values
11	[Implement]	If the results are good, implement the model

**Table 2:** Degree of influence.

Degree of influence	Determination of influence
Very low	0
Low	1
Normal	2
High	3
Very high	4
Extremely high	5

**Table 3:** Effort comparisons.

Traditional FP	FFPA-PM	FFPA-PSR	Real effort
44.37	45.57	46.25	46.43
50.12	51.32	52.87	53.10
51.32	52.57	53.54	53.92
46.35	48.16	49.24	49.45
48.10	49.96	50.34	50.67
45.32	46.62	47.92	48.10
46.45	48.26	49.34	49.54
50.22	51.42	52.97	53.20
48.20	50.10	51.44	51.87
44.44	45.62	47.35	47.89

**Table 4:** Performance comparison.

Model	MMRE	PRED	Accuracy	Improved accuracy
Traditional FPA	0.42	0.5	87.16	NA
FFPA-PM	0.27	0.7	96.45	9.29%
FFPA-PSR	0.23	0.9	98.92	11.76%
